# Combination therapy with rifaximin and lactulose in hepatic encephalopathy: A systematic review and meta-analysis

**DOI:** 10.1371/journal.pone.0267647

**Published:** 2022-04-26

**Authors:** Jian Fu, Yi Gao, Li Shi

**Affiliations:** Department of Infection, Hainan General Hospital, Hainan Affiliated Hospital of Hainan Medical University, Haikou, China; Cliniques Universitaires Saint-Luc, BELGIUM

## Abstract

Rifaximin and lactulose are widely used in patients with hepatic encephalopathy (HE); however, data on whether the combined use of rifaximin and lactulose could yield additional benefits for patients with HE are limited and inconclusive. We conducted a systematic review and meta-analysis of randomized controlled trials (RCTs) to determine the treatment effectiveness of rifaximin plus lactulose versus lactulose alone in patients with HE. Electronic databases (PubMed, Embase, Cochrane Library, and China National Knowledge Infrastructure) were searched for eligible RCTs from their inception until November 2020. Relative risks (RRs) with 95% confidence intervals (CIs) were applied to calculate pooled effect estimates for the treatment effectiveness of rifaximin plus lactulose versus lactulose alone by using the random-effects model. Sensitivity, subgroup, and publication bias analyses were also performed. We included 7 RCTs enrolling 843 patients with HE. We noted that the use of rifaximin plus lactulose was associated with an increased incidence of effective rate than lactulose alone (RR, 1.30; 95% CI, 1.10–1.53; *P* = 0.002). Moreover, the use of rifaximin plus lactulose was associated with a reduced risk of mortality as compared with lactulose alone (RR, 0.57; 95% CI, 0.41–0.80; *P* = 0.001). This study found that the use of rifaximin in combination with lactulose could provide additional benefits in terms of increased effective rate and decreased mortality than lactulose alone in patients with HE.

## Introduction

Hepatic encephalopathy (HE) is induced by metabolic disorders which cause severe liver disease and lead to the dysfunction of the central nervous system [1, 2]. The main clinical manifestations of HE include disturbances of consciousness, behavioral disorders, and coma [3]. Compared with variceal bleeding and ascites, HE alone is associated with the worst outcome, with reported 1-year mortality as high as 64%, which confers a damning prognosis [4]. Even in its subclinical or covert state, it triggers a profound negative impact on the functional capability and quality of life of both patients and their caregivers [5, 6]. The prevalence of HE is relatively high, and should be considered a common complication of severe liver disease, including hepatitis caused by a variety of viruses, cirrhosis, and liver cancer [7]. Nowadays, the mechanisms of HE pathogenesis include ammonia poisoning, inflammatory response to injury, amino acid imbalance, and pseudo neurotransmitter replace normal neurotransmitter [8–11]. HE is regarded as an important predictor associated with poor prognosis [12]. Therefore, improving HE is of great importance in clinical practice to improve patient prognosis.

Nonabsorbable disaccharides are considered as the first-line treatment option for patients with HE. Lactulose reduces the concentration of aminogenic substrates in the intestinal lumen and pH in the colon through the production of organic acids by bacterial fermentation, and the osmotic cathartic mechanism [13, 14]. Rifaximin, an oral antimicrobial agent, was approved for the prevention and treatment of HE by the US Food and Drug Administration in 2010 [15]. Rifaximin should be used in combination with lactulose as a treatment strategy in patients with HE [16]. However, it remains unclear whether the combined use rifaximin and lactulose could provide additional benefits over lactulose alone in patients with HE. Therefore, we performed a systematic review and meta-analysis of randomized controlled trials (RCTs) to assess the effectiveness of rifaximin plus lactulose versus lactulose alone in patients with HE.

## Materials and methods

### Data sources, search strategy, and selection criteria

This study was conducted and the results reported according to the Preferred Reporting Items for Systematic Reviews and Meta-Analysis guidelines [17]. We included RCTs comparing the effectiveness of rifaximin plus lactulose versus lactulose alone in patients with HE. No restrictions were placed on publication language and status. We systematically searched the following databases: PubMed, Embase, Cochrane Library, and China National Knowledge Infrastructure from their inception throughout November 2020, and the following search terms were used as the Medical Subject Heading and text words: “hepatic encephalopathy,” “rifaximin,” and “lactulose.” We also manually searched the reference lists of retrieved studies to identify any other studies that met the inclusion criteria.

Two reviewers independently conducted the literature search and study selection, and any disagreement was resolved by discussion until mutual consensus was reached. A study was included if it met the following criteria: 1) patients: HE; 2) intervention: rifaximin plus lactulose; 3) control: lactulose alone; 4) outcome: the effective rate and mortality; effective rate was defined as improved clinical and neurological status, or significant decrease in HE; and 5) study design: RCT. This study did not contain any human participants, and the requirements for ethics approval and informed consent were not applicable.

### Data collection and quality assessment

Two reviewers independently abstracted data and assessed quality. Any inconsistencies between the reviewers were discussed with an additional reviewer until a consensus was reached. The collected items included the name of the first author, publication year, country, sample size, mean age, proportion of male participants, HE type, etiology, severity, intervention, control, treatment duration, follow-up, and reported outcomes. The quality of each RCT was assessed using the Jadad scale, which assesses randomization, blinding, allocation concealment, withdrawals and dropouts, and the use of intention-to-treat analysis [18]. The scoring system for Jadad scale ranges from 0 to 5, and the studies with scores of 4 or 5 were considered of high quality.

### Statistical analysis

The incidences of effective rate and mortality between rifaximin plus lactulose and lactulose alone in patients with HE were assigned as categorical data. Relative risks (RRs) with 95% confidence intervals (CIs) were calculated before data pooling. Then, the pooled results were calculated using the random-effects model, which considers the underlying differences among included studies [19, 20]. Heterogeneity across included studies was assessed with the *I*^*2*^ and Q statistic, and significant heterogeneity was defined as *I*^*2*^ >50.0% or *P* <0.10 [21, 22]. Sensitivity analysis for effective rate and mortality was also conducted to assess the robustness of pooled results by sequential exclusion of individual studies [23]. Subgroup analysis for effective rate and mortality were also conducted based on country, mean age, male proportion, HE type, etiology, and study quality, and the difference between subgroups was assessed by using the interaction *P* test [24]. Publication bias for effective rate and mortality were also assessed by using funnel plots, Egger and Begg tests results [25, 26]. All statistical tests were two sided, and *P* values <0.05 were considered to be statistically significant. STATA software (version 10.0; StataCorp, Texas, USA) was applied to conduct all statistical analyses in this study.

## Results

### Literature search

**Fig 1:**
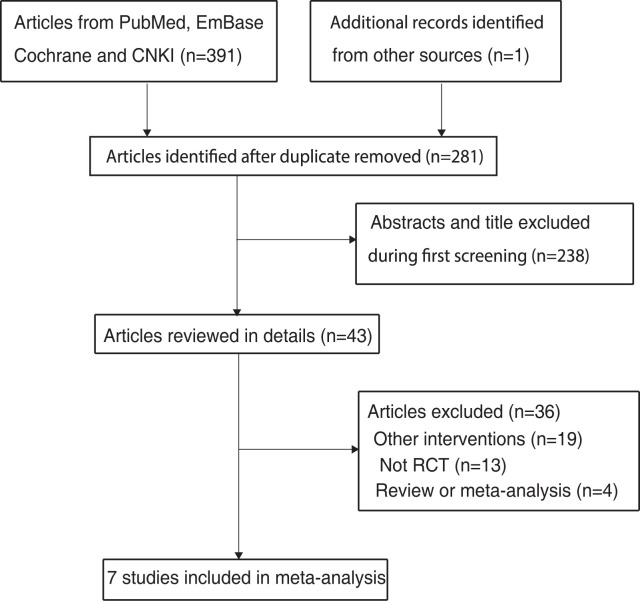
The PRISMA flowchart for the literature search and study selection.

The PRISMA flowchart of the study selection process is shown in Fig 1. In the initial electronic search, we found 391 eligible articles, and 281 articles were retained after duplicate records were removed. A total of 238 studies were further excluded due to irrelevancy. The remaining 43 studies were retrieved for further full-text evaluations, and 36 studies were excluded due to the following causes: other interventions (n = 19); not RCT (n = 13); review or meta-analysis (n = 4). Reviewing the reference lists of the remaining 7 studies did not reveal any new eligible studies. Finally, 7 RCTs were selected for our final meta-analysis [27–33].

### Study characteristics

The baseline characteristics of included studies and recruited patients are summarized in Table 1. A total of 843 patients with HE were included, and the sample size ranged from 62 to 200. Three RCTs were conducted in Pakistan, 2 in India, and the remaining 2 in China. Four trials included patients with HE, and the remaining 3 trials included patients with overt HE. Two of the included trials were of high quality and scored 4 on the Jadad score, 3 scored 3, and the remaining 2 scored 2.

**Table 1:** The baseline characteristics of included studies and patients.

Study	Country	Sample size	Mean age (years)	Male (%)	HE type	Etiology	Severity	Intervention	Control	Treatment duration	Follow-up	Study quality
Gao 2012 [24]	China	62 (31/31)	59.5	66.1	HE	Cirrhosis	Not assigned	Rafaximin 550 mg and lactulose 60 ml daily	Lactulose 60 ml daily	10–15 days	10–15 days	2
Sharma 2013 [25]	India	120 (63/57)	39.4	74.2	Overt HE	Alcohol: 72; HBV: 32; HCV: 10; other: 29	CPT: 9.6; MELD: 24.3; HE grade (2/3/4): 22/40/58	Rafaximin 1,200 mg and lactulose 90–180 ml daily	Lactulose 90–180 ml daily	≤ 10 days	In-hospital	4
Gill 2014 [26]	Pakistan	200 (100/100)	40.0	70.0	Overt HE	Cirrhosis	HE grade (2/3/4): 60/70/70	Rafaximin 1,100 mg and lactulose 60–180 ml daily	Lactulose 60–180 ml daily	10 days	In-hospital	3
Muhammad 2014 [27]	Pakistan	160 (80/80)	41.0	55.6	HE	Cirrhosis	HE grade (1/2/3/4): 3/29/51/77	Rafaximin 1,100 mg and lactulose 90 ml daily	Lactulose 90 ml daily	7 days	7 days	2
Hasan 2018 [28]	India	91 (45/46)	44.9	81.3	Overt HE	Alcohol: 81; others: 10	Not assigned	Rafaximin 1,200 mg and lactulose 60–120 ml daily	Lactulose 60–120 ml daily	≤ 10 days	10 days	4
Butt 2018 [29]	Pakistan	130 (65/65)	56.1	53.1	HE	Decompensated chronic liver disease	HE grade (2/3/4): 43/49/38	Rafaximin 1,100 mg and lactulose 90 ml daily	Lactulose 90 ml daily	10 days	10 days	3
Fan 2019 [30]	China	80 (40/40)	44.6	76.3	HE	Cirrhosis	HE grade (1/2/3): 17/27/36	Rafaximin 800 mg and lactulose 30 ml daily	Lactulose 30 ml daily	7 days	7 days	3

### Meta-analysis

**Fig 2:**
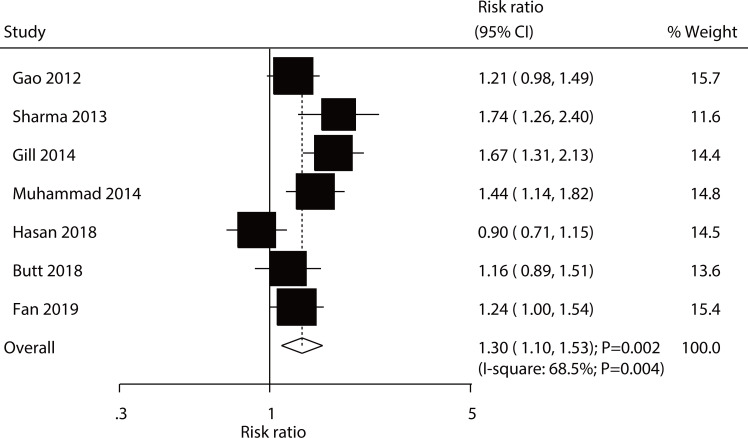
Effect of rifaximin plus lactulose versus lactulose alone on the incidence of effective rate.

**Fig 3:**
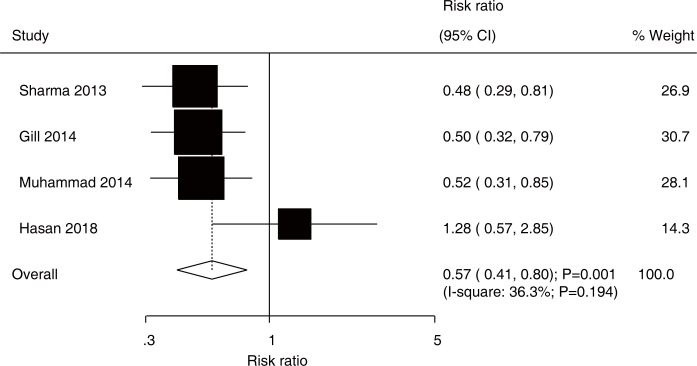
Effect of rifaximin plus lactulose versus lactulose alone on the risk of mortality.

After pooling data from all included studies, we noted that the use of rifaximin plus lactulose was associated with an increased incidence of effective rate as compared with lactulose alone in patients with HE (RR, 1.30; 95% CI, 1.10–1.53; *P* = 0.002; Fig 2). Included trails were heterogeneous (*I*^*2*^ = 68.5%; *P* = 0.004). Furthermore, treatment with rifaximin plus lactulose was associated with a reduced risk of mortality as compared with lactulose alone (RR, 0.57; 95% CI, 0.41–0.80; *P* = 0.001; Fig 3), and no heterogeneity was detected across included trials (*I*^*2*^ = 36.3%; *P* = 0.194).

### Sensitivity and subgroup analyses

**Fig 4:**
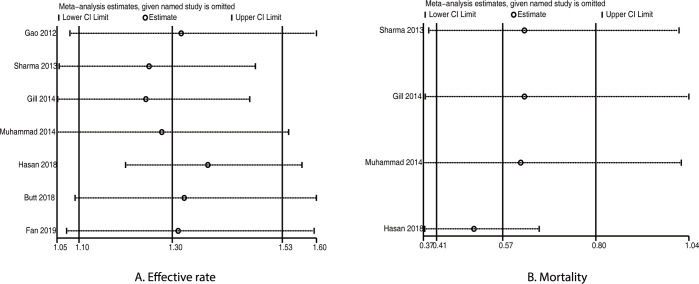
Sensitivity analyses for effective rate and mortality.

Sensitivity analyses were also performed for effective rate and mortality and are presented in Fig 4. The pooled data for effective rate were robust and this did not change by sequential exclusion of individual trials, whereas the pooled data for mortality were variable because of the smaller number of included trials. Although the significant differences between rifaximin plus lactulose and lactulose alone for the incidence of effective rate were observed in most subgroups, we noted that treatment with rifaximin plus lactulose was not associated with the incidence of effective rate when: pooled studies were conducted in India, male proportion was ≥70.0%, patients had overt HE, patients had other etiologies, or studies were of high quality (Table 2). Moreover, the risk of mortality was significantly reduced in patients treated with rifaximin plus lactulose when: pooled studies were conducted in Pakistan, mean age was <50.0 years, male proportion was <70.0%, patients had HE, the etiology was cirrhosis, or studies were of low quality (Table 2).

**Table 2:** Subgroup analyses for effective rate and mortality.

Outcomes	Factors	Subgroup	RR and 95%CI	P value	Heterogeneity (%)	P value for heterogeneity	P value between subgroups
Effective rate	Country	China	1.22 (1.05–1.42)	0.009	0.0	0.860	0.153
		Pakistan	1.41 (1.16–1.73)	0.001	49.6	0.138	
		India	1.24 (0.63–2.45)	0.534	91.2	0.001	
	Mean age (years)	≥ 50.0	1.19 (1.01–1.40)	0.041	0.0	0.787	0.287
		< 50.0	1.35 (1.07–1.71)	0.012	77.6	0.001	
	Male proportion (%)	≥ 70.0	1.33 (0.98–1.81)	0.067	82.9	0.001	1.000
		< 70.0	1.27 (1.11–1.45)	0.001	0.0	0.398	
	HE type	HE	1.26 (1.12–1.41)	< 0.001	0.0	0.599	0.763
		Overt HE	1.37 (0.87–2.15)	0.173	88.3	< 0.001	
	Etiology	Cirrhosis	1.37 (1.17–1.60)	< 0.001	46.8	0.130	0.108
		Other	1.20 (0.83–1.74)	0.325	81.5	0.004	
	Study quality	High	1.24 (0.63–2.45)	0.534	91.2	0.001	0.251
		Low	1.33 (1.16–1.52)	< 0.001	37.8	0.170	
Mortality	Country	Pakistan	0.51 (0.36–0.71)	< 0.001	0.0	0.931	0.399
		India	0.75 (0.29–1.93)	0.549	75.0	0.046	
	Mean age (years)	≥ 50.0	-	-	-	-	-
		< 50.0	0.57 (0.41–0.80)	0.001	36.3	0.194	
	Male proportion (%)	≥ 70.0	0.62 (0.37–1.02)	0.059	56.5	0.101	0.731
		< 70.0	0.52 (0.31–0.85)	0.009	-	-	
	HE type	HE	0.52 (0.31–0.85)	0.009	-	-	0.731
		Overt HE	0.62 (0.37–1.02)	0.059	56.5	0.101	
	Etiology	Cirrhosis	0.51 (0.36–0.71)	< 0.001	0.0	0.931	0.399
		Other	0.75 (0.29–1.93)	0.549	75.0	0.046	
	Study quality	High	0.75 (0.29–1.93)	0.549	75.0	0.046	0.399
		Low	0.51 (0.36–0.71)	< 0.001	0.0	0.931	

### Publication bias

**Fig 5:**
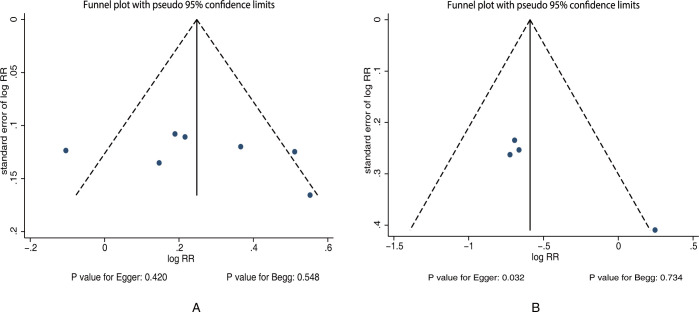
Funnel plot for effective rate and mortality. (A) Effective rate. (B) Mortality.

There was no significant publication bias for effective rate (*P* value for Egger: 0.420; *P* value for Begg: 0.548; Fig 5A). Although no significant publication bias for mortality was found in the Begg test, the Egger test indicated potentially significant publication bias (*P* value for Egger: 0.032; *P* value for Begg: 0.734; Fig 5B). The pooled conclusion did not change after adjustment for potential publication bias by using the trim and fill method [34].

## Discussion

The current systematic review and meta-analysis compared the effectiveness of rifaximin plus lactulose with lactulose alone on the incidences of effective rate and mortality in patients with HE. This study included 7 RCTs and enrolled 843 patients with HE assessing a broad range of patient characteristics. This study found that the use of rifaximin plus lactulose could provide additional benefits on the incidences of effective rate and mortality than lactulose alone. Subgroup analyses found that the beneficial effects of rifaximin plus lactulose were mainly present in the following subgroups: studies conducted in China and Pakistan, mean age <50.0 years, male proportion <70.0%, patients with HE, cirrhosis as the etiology, or low-quality studies.

A meta-analysis by Eltawil et al including 12 RCTs found rifaximin could provide effects equivalent to those of disaccharides or other oral antibiotics, and had a better safety profile for patients with HE [35]. However, this study did not assess the treatment effectiveness of rifaximin plus lactulose, and the stratified analyses were mainly based on control. Wang et al conducted a meta-analysis of 5 RCTs and 5 observational studies and found rifaximin plus lactulose could yield additional benefits on effective rate and mortality. The pooled analyses of 5 RCTs found similar effectiveness of rifaximin plus lactulose in patients with HE [36]. However, stratified analyses according to study and patient characteristics were not provided. We therefore conducted an updated meta-analysis of RCTs to determine the effectiveness of rifaximin plus lactulose versus lactulose alone in patients with HE.

The summary result of our study indicated that rifaximin plus lactulose versus lactulose alone was associated with an increased incidence of effective rate. Most of the included trials reported similar conclusions or trends. However, Hasan et al found the use of lactulose alone could improve the neurological status in patients with overt HE [31]. The potential reason for this could be that intestinal bacteria overmultiply and intestinal dynamics are disturbed in patients with cirrhosis, which could induce an increase in the levels of inflammatory markers and aggravating liver damage. The use of rifaximin could inhibit the bacterial polymerase and block the transcription process of bacterial RNA, which could hinder the synthesis of bacterial protein and reduce the production of ammonia.

We noted the use of rifaximin plus lactulose could protect against the risk of mortality as compared with lactulose alone in patients with HE. The potential reason for this could be that rifaximin plus lactulose might reduce sepsis-related death because of a decrease in the blood levels of a gut-related endotoxin [28]. Moreover, the use of rifaximin plus lactulose might reduce the progression of HE and its severity [37]. However, in this study, there was only 1 study reporting adverse events between rifaximin plus lactulose and lactulose alone [28], which requires further verification in a large-scale RCT.

In subgroup analyses, the benefits between rifaximin plus lactulose and lactulose alone were found mainly in the following subgroups: studies conducted in China and Pakistan, mean age <50.0 years, male proportion <70.0%, patients with HE, cirrhosis as the etiology, or low-quality studies. Although the effects of rifaximin plus lactulose in India mainly affected by the study conducted by Hasan et al [31], and only 2 trials were conducted in India. Moreover, younger patients, patients with mild HE, and those with cirrhosis as the etiology could benefit more from rifaximin plus lactulose. Of note, women might benefit more than men, which might be related to their dietary lifestyle and the severity of disease. Finally, the quality of the study was correlated with the reliability of the pooled data, and the conclusions of this study warrant verification in further high-quality studies.

Several limitations of this study should be acknowledged. First, all of the included studies were conducted in China, Pakistan, and India, thus the conclusions were limited to reflect the exact effect in other countries. Second, the heterogeneity among included studies did not allow full interpretation by sensitivity and subgroup analyses. Third, only 4 of included trials reported the risk of mortality between rifaximin plus lactulose and lactulose alone, and the results of stratified analyses were variable. Fourth, this study has inherent limitations of traditional meta-analyses, including the use of pooled data and inevitable publication bias.

## Conclusion

In conclusion, this study found the use of rifaximin plus lactulose could increase the incidence of effective rate, and reduce the risk of mortality when compared with lactulose alone in patients with HE. Therefore, rifaximin plus lactulose should be applied in clinical practice, especially in China, Pakistan, and India. A large-scale RCT is needed to compare the long-term effectiveness of rifaximin plus lactulose with lactulose alone in patients with HE.
